# Enhancing the Therapeutic Potential of Sulfamidase for the Treatment of Mucopolysaccharidosis IIIA

**DOI:** 10.1016/j.omtm.2019.10.009

**Published:** 2019-10-28

**Authors:** Nicolina Cristina Sorrentino, Vincenzo Cacace, Maria De Risi, Veronica Maffia, Sandra Strollo, Novella Tedesco, Edoardo Nusco, Noemi Romagnoli, Domenico Ventrella, Yan Huang, Nan Liu, Susan L. Kalled, Vivian W. Choi, Elvira De Leonibus, Alessandro Fraldi

**Affiliations:** 1 Telethon Institute of Genetics and Medicine, Via Campi Flegrei 34, Pozzuoli, Naples, Italy; 2 Department of Veterinary Medical Sciences, University of Bologna, Via Tolara di Sopra 50, Ozzano dell’Emilia, Bologna, Italy; 3 Takeda Pharmaceuticals, Cambridge, MA, USA; 4 Institute of Cellular Biology and Neurobiology (IBCN), National Research Council (CNR), Via Ramarini 32, Monterotondo, Rome, Italy; 5 Department of Translational Medicine, University of Naples “Federico II,” Naples, Italy

## Abstract

Mucopolysaccharidosis type IIIA (MPS-IIIA) is a lysosomal storage disorder (LSD) caused by inherited defect of sulfamidase, a lysosomal sulfatase. MPS-IIIA is one of the most common and severe forms of LSDs with CNS involvement. Presently there is no cure. Here we have developed a new gene delivery approach for the treatment of MPS-IIIA based on the use of a modified version of sulfamidase expression cassette. This cassette encodes both a chimeric sulfamidase containing an alternative signal peptide (sp) to improve enzyme secretion and sulfatase-modifying factor 1 (SUMF1) to increase sulfamidase post-translational activation rate. We demonstrate that improved secretion and increased activation of sulfamidase act synergistically to enhance enzyme biodistribution in wild-type (WT) pigs upon intrathecal adeno-associated virus serotype 9 (AAV9)-mediated gene delivery. Translating such gene delivery strategy to a mouse model of MPS-IIIA results in a rescue of brain pathology, including memory deficit, as well as improvement in somatic tissues. These data may pave the way for developing effective gene delivery replacement protocols for the treatment of MPS-IIIA patients.

## Introduction

Mucopolysaccharidosis type IIIA (MPS-IIIA) is one of the most common and severe forms of neurodegenerative lysosomal storage disorders (LSDs).^1^^,^^2^ It is caused by inherited defect of the sulfamidase (SGSH), a soluble lysosomal enzyme belonging to the family of sulfatases,^3^ and leads to the accumulation of heparan sulfate in cell, particularly within the CNS. Presently, there are no treatments available to treat the CNS in MPS-IIIA patients. Gene delivery aimed at correcting defective hydrolytic lysosomal defects represents the most promising replacement strategy for the CNS treatment of MPS-IIIA, as well as other MPSs with similar causes, because of its potential for a one-time treatment.^4^

Among viral vectors used for gene transfer, adeno-associated viral (AAV) vectors are most commonly utilized for *in vivo* gene transfer because they are safe, provide significantly long transgene expression, and may be generated with variable serotypes allowing efficient delivery of therapeutic genes to different target tissues.^5^^,^^6^ To date, several therapeutic approaches have been designed and developed based on AAV-mediated gene delivery of SGSH using different routes of administration to reach the CNS.^7^, ^8^, ^9^, ^10^, ^11^, ^12^ Although all of these approaches showed potential benefits in preclinical animal models, the effective therapeutic application of these protocols in the clinical management of MPS-IIIA patients is challenging because of the difficulty in achieving widespread distribution of the corrective enzyme in the CNS and in maintaining therapeutic threshold levels of them in targeted cells.^13^ Phase I/II clinical trials based on either AAV9-mediated systemic (ClinicalTrials.gov: NCT02716246) or AAVrh10-mediated intra-parenchymal delivery of the *SGSH* gene are ongoing (ClinicalTrials.gov: NCT03612869). Although these approaches are promising, they also show drawbacks. Indeed, the first approach suffers from potential toxicity because of the high doses of systemically delivered AAV9 vectors required for efficacy,^14^ whereas the second approach is invasive and preliminary efficacy data did not show evident improvement of CNS pathology, likely also due to the inefficient targeting of CNS regions at any significant distance from the injection sites.^15^

Therefore, a major medical need in the clinical care of MPS-III patients is to overcome these limitations and develop safe and minimally invasive gene transfer approaches with improved CNS transduction.

Enhancing the therapeutic potential of sulfamidase, along with developing tools for efficient and safe CNS targeting, may represent a novel area of intervention to improve CNS therapy in MPS-IIIA patients. Intra-cerebrospinal fluid (CSF) administration allows circulating virus to reach a large CNS surface area, and at the same time provides access to visceral organs due to the leakage of vector into the bloodstream.^9^^,^^16^^,^^17^ Among different AAV serotypes, serotype 9 has been shown to have a broad tropism, including neurons and astrocytes, when delivered either via CSF or through intravenous injection due to its capability to cross the blood-brain barrier (BBB).^18^^,^^19^ We recently demonstrated that AAV9 outperforms all other AAV serotypes tested in CNS transduction efficiency when administered via CSF even in large-animal models.^20^

Lysosomal hydrolases, including SGSH, can be secreted and taken up by surrounding cells.^21^ Such cross-correction capability makes gene replacement protocols potentially more effective because transduced cells (factory cells) may also correct non-transduced cells. In a previous study we demonstrated that replacing the signal peptide (sp) of sulfamidase with that belonging to another lysosomal hydrolase that is highly secreted, the iduronate 2-sulfatase (IDS), strongly improved the efficiency of SGSH secretion from multiple cell types both *in vivo* and *in vitro*, a property that enhanced the cross-correction capability of transduced cells upon gene delivery.^8^

To be fully active, all sulfatases, including the SGSH, must be activated through the generation of the catalytic residue C-alpha-formylglycine (FGly). This post-translational modification is accomplished in the endoplasmic reticulum by the FGly-generating enzyme (FGE) encoded by the sulfatase-modifying factor 1 (SUMF1) gene.^22^ SUMF1 is both an essential and a limiting factor for sulfatases. Co-expression of SUMF1 with sulfatases results in a synergistic increase of sulfatase activity.^22^^,^^23^ Here we have generated a potentiated SGSH expression cassette by combining enhanced secretion efficiency of SGSH with improved SUMF1-mediated enzyme activation capacity and explored its therapeutic potential upon intra-CSF AAV9-mediated gene delivery. Our results demonstrate that by using this strategy it is possible to achieve a superior SGSH CNS biodistribution in a large-animal model (wild-type [WT] pigs) and to fully rescue the CNS phenotype in a mouse model of MPS-IIIA.

## Results

### Usage of a SGSH Expression Cassette with Enhanced Enzyme Secretion and Activation Improved Enzyme Biodistribution in the CNS of Pigs upon Intra-CSF AAV9-Mediated Gene Delivery

**Figure 1:**
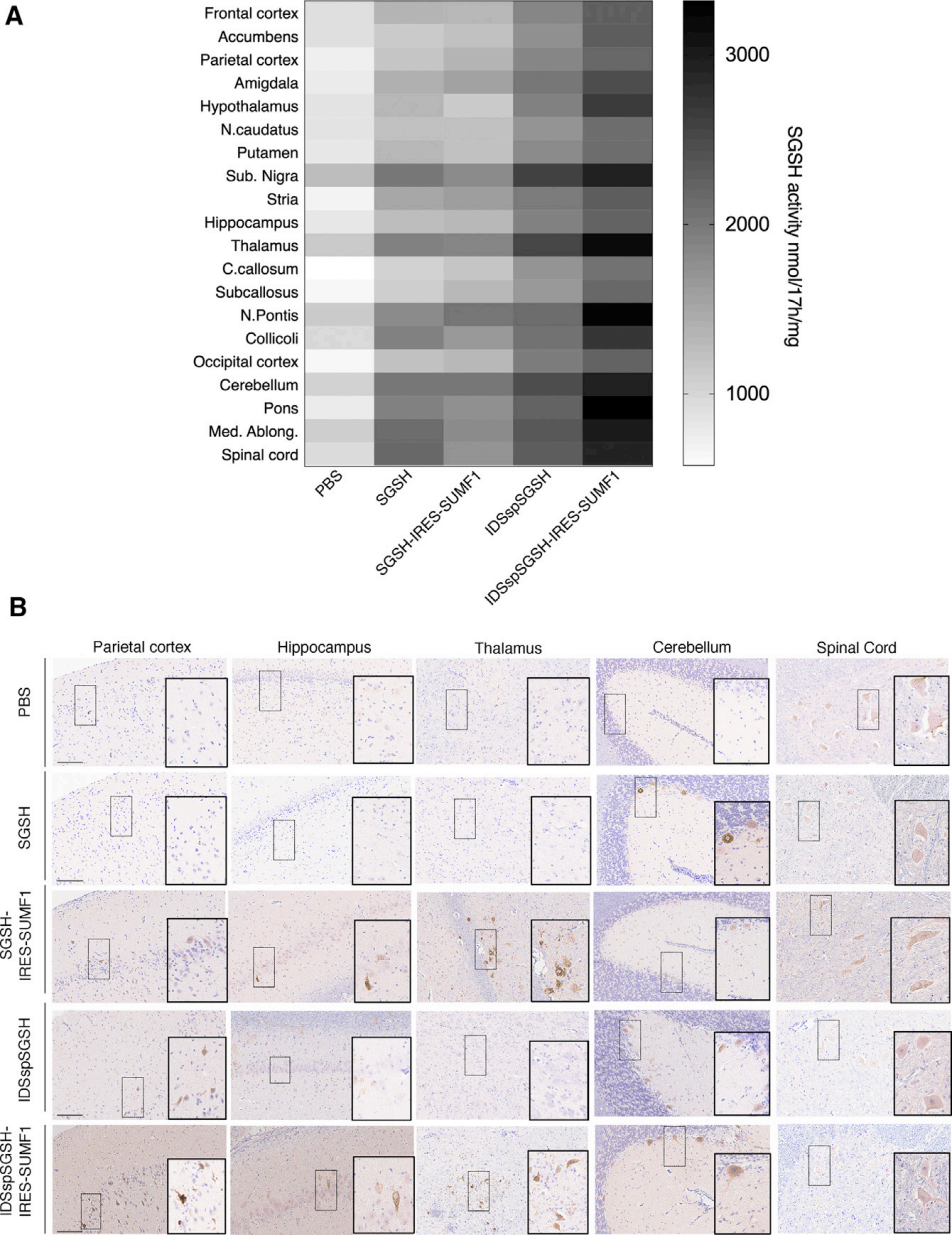
Sulfamidase-Specific Activity in the CNS of Pigs Injected with AAV9 Vectors Carrying Different SGSH Expression Cassettes (A) WT pigs at P60 were injected via cisterna magna with 4.5 × 10^12^ GCs/kg of AAV9 encoding different human SGSH expression cassettes under the CMV promoter: WT SGSH, *IDSspSGSH*, *SGSH*-IRES-*SUMF1*, and *IDSspSGSH*-IRES-*SUMF1*. At 1 month after injection, sulfamidase-specific activity (nmol/17 h/mg of protein) was measured in the indicated 20 different areas of the CNS (F. cortex and O. cortex mean frontal and occipital cortex, respectively) and compared with control WT pigs treated with PBS. n = 5 animals for each group. Data represent mean ± SEM. (B) IHC staining with anti-human sulfamidase in main representative CNS areas of injected WT pigs. Scale bars: 300 μm.

In order to improve the biodistribution potential of the SGSH enzyme, we generated a bicistronic SGSH expression cassette containing a chimeric SGSH bearing the iduronate 2-sulfatase signal peptide (IDSsp) and the gene encoding SUMF1 (*IDSspSGSH*-IRES-*SUMF1*). The SGSH biodistribution in the CNS triggered by *IDSspSGSH*-IRES-*SUMF1* expression was then evaluated upon intra-CSF AAV-mediated delivery in WT pigs (*Sus Scrofa* white model). Thirty days after birth (P30), WT pigs were intra-CSF injected via cisterna magna with 4.5 × 10^12^ GCs/kg of AAV9 encoding *IDSspSGSH*-IRES-*SUMF1* under the CMV promoter. As control, pigs were injected with AAV9 encoding for only either WT SGSH (AAV9-*SGSH*), IDSsp-modified SGSH (AAV9-*IDSspSGSH*), or bicistronic cassette containing SUMF1 together with WT SGSH (AAV9-*SGSH*-IRES-*SUMF1*). One month after injection, 20 CNS regions covering the main representative areas of the brain and the spinal cord were collected for both SGSH enzyme activity evaluation and immunohistochemistry (IHC) analysis. The expression of highly secreted forms of SGSH (*IDSspSGSH* and *IDSspSGSH*-IRES-*SUMF1* cassettes) resulted in a remarkable and significant increase of the specific enzyme activity over the CNS as compared with control (pigs injected with unmodified SGSH constructs: *SGSH* and *SGSH*-IRES-*SUMF1*) (Figure 1A; Table S1). Interestingly, the enhancing effect of SUMF1 was statistically significant only when SUMF1 was co-expressed with IDSsp-modified SGSH (*IDSspSGSH*-IRES-*SUMF1*) and not when it was co-expressed with unmodified SGSH (*SGSH*-IRES-*SUMF1*) (Figure 1A; Table S1). Immunostaining analysis against exogenous human sulfamidase revealed a very strong SGSH expression in multiple brain areas of pigs injected with *IDSspSGSH*-IRES-*SUMF1*, which indicated that the increased enzymatic activity is associated with a strong SGSH protein signal in the same brain areas of interest (Figure 1B). Our results showed that combining improved secretion and enhanced SUMF1-mediated enzymatic activation gave rise to a synergistic effect on SGSH activity in the CNS of a large-animal model.

### AAV9-Mediated Intra-CSF Delivery of *IDSspSGSH-*IRES*-SUMF1* Resulted in a Strong and Sustained SGSH Activity in the Brain of *Sgsh*^−/−^ Mice

To evaluate the potential for therapeutic effectiveness of intra-CSF AAV9-mediated delivery of *IDSspSGSH*-IRES-*SUMF1*, we used the B6Cg-*Sgsh*^(mps3a/PstJ)^ mouse (*Sgsh*^−/−^), a spontaneous MPS-IIIA mouse model largely used in preclinical studies of this disease.^24^ Initially, we performed a short-term comparative study in a small cohort of *Sgsh*^−/−^ mice (hereinafter referred to as MPS-IIIA mice) to evaluate the efficacy of the *IDSspSGSH*-IRES-*SUMF1* expression cassette on a background of disease-associated enzymatic activity (MPS-IIIA levels in this model show 3%–5% of normal SGSH activity). P60 MPS-IIIA mice were intraventricularly (i.c.v.) injected with 5.4 × 10^12^ GCs/kg of AAV9 vectors encoding WT *SGSH*, *IDSspSGSH*, or *IDSspSGSH*-IRES-*SUMF1*. One month after injection, we found a significant increase in SGSH activity in brain sections from the *IDSspSGSH*-injected MPS-IIIA mice and a further significant increase in enzymatic activity upon the administration of AAV9-*IDSspSGSH*-IRES-*SUMF1* (until to 50% of WT levels) (Figure S1). These results confirmed the data obtained in pigs showing enhanced therapeutic potential of the *IDSspSGSH*-IRES-*SUMF1* expression cassette.

**Figure 2:**
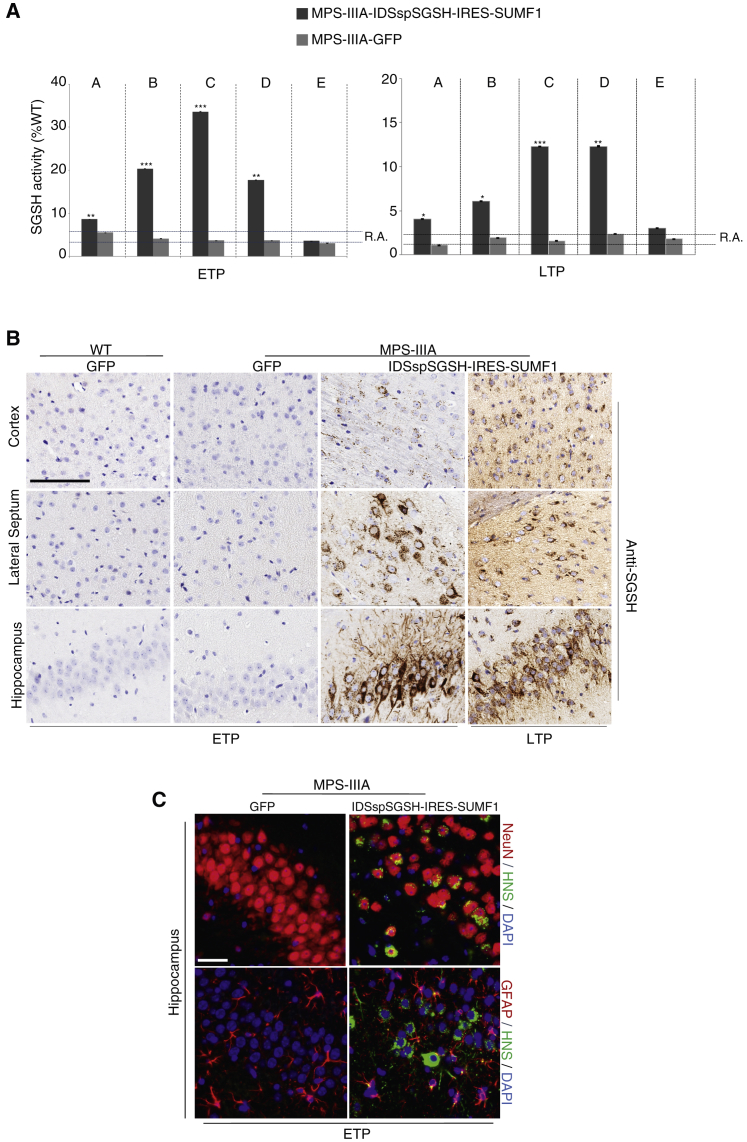
CNS Transduction in MPS-IIIA Mice Injected with AAV9 Bearing *IDSspSGSH*-IRES-*SUMF1* Transgene (A) P60 MPS-IIIA mice were i.c.v. injected with 4.5 × 10^12^ GCs/kg of AAV9 encoding either IDSspSGSH and SUMF1 or GFP. Five different slices covering the main CNS regions (A–E; as described in Figure S1) were collected at both 1 (ETP) and 7 months (LTP) after injection. Sulfamidase activity was measured in these areas and expressed as percentage of the activity found in age-matched WT injected with AAV9 encoding *GFP*. n = 6–7 animals for each group. Data represent mean ± SEM. *p < 0.05, **p < 0.01, ***p < 0.00 versus MPS-IIIA GFP-treated. One-way ANOVA followed by Tukey’s post hoc test. (B) Anti-human sulfamidase immunostaining in representative brain regions of MPS-IIIA mice was shown at both ETP and LTP upon i.c.v. injection. Scale bar: 100 μm. (C) Co-immunofluorescence analysis of human sulfamidase together with either GFAP (astroglial marker) or NeuN (neuronal marker) in the hippocampus of MPS-IIIA mice injected with AAV9 encoding *GFP* or *IDSspSGSH*-IRES-*SUMF1* expression cassette. Scale bar: 50 μm.

We then performed an efficacy study in a larger cohort of MPS-IIIA mice in which AAV9 encoding *IDSspSGSH*-IRES-*SUMF1* was delivered i.c.v. Following 1 (early time point [ETP]) or 7 months (late time point [LTP]) after injection, the enzyme biodistribution and the pathological phenotype were analyzed. Consistent with previous results (Figure S1), a significant increase of SGSH activity in the brain of injected animals with peaks of 35% of WT levels was observed at ETP (Figure 2A). Importantly, such increased activity was sustained at LTP (25% of WT levels) (Figure 2A). Increased SGSH activity correlated with both the increase in SGSH protein expression (Figure S2A; Figure 2B) and the number of GCs detected in the brain (Figure S2B). Co-immunolabeling analysis of cell-specific transduction in the brain samples of MPS-IIIA mice i.c.v. injected with AAV9-encoding *IDSspSGSH*-IRES-*SUMF1* showed that SGSH signal co-localized with both NeuN (neuronal nuclei protein) and glial fibrillary acidic protein (GFAP) (astroglial) markers in several brain regions, with NeuN co-localization being prevalent over GFAP co-localization (Figure 2C).

### Rescue of Storage Pathology, Lysosomal Enlargement, and Neuroinflammation in MPS-IIIA Mice Treated with AAV9-*IDSspSGSH-*IRES*-SUMF1*

**Figure 3:**
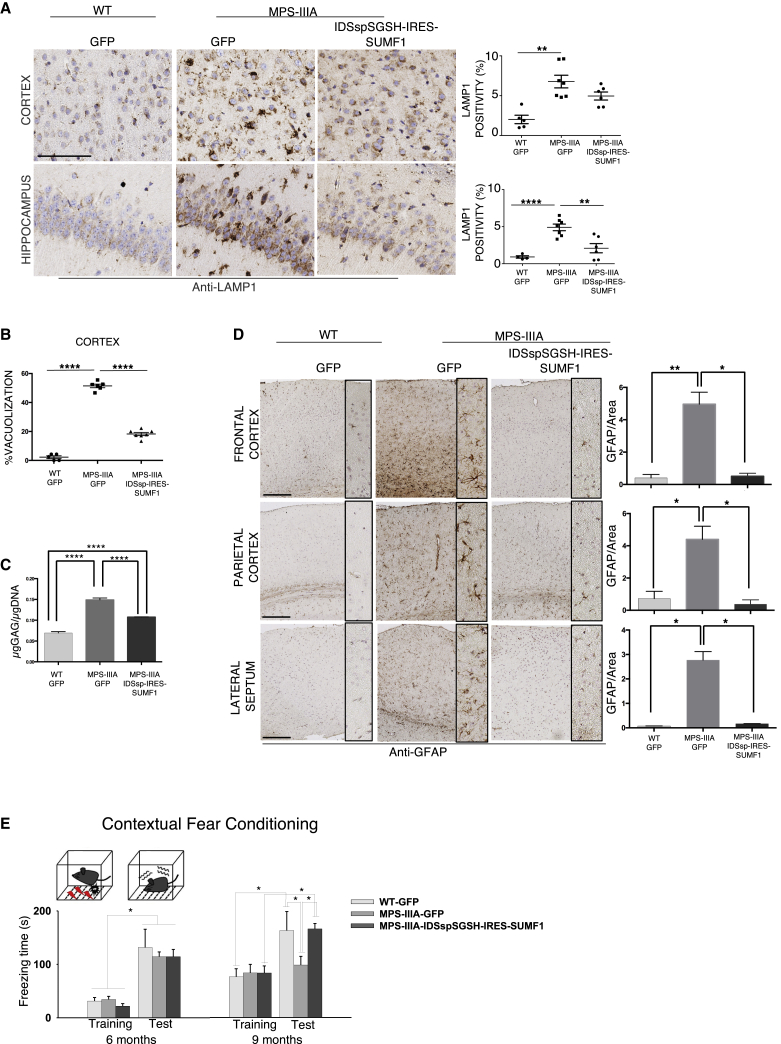
Rescue of Storage Pathology, Inflammation, and Memory Impairment in MPS-IIIA Mice Injected with AAV9 Encoding IDSspSGSH-IRES-SUMF1 (A) LAMP1 immunostaining and relative quantification in the cortex and hippocampus of MPS-IIIA mice i.c.v. injected with AAV9 encoding *IDSspSGSH*-IRES-*SUMF1* collected at LTP. Age-matched WT and MPS-IIIA mice i.c.v. injected with AAV9 encoding GFP were used as controls. Scale bar: 100 μm. n = 5–7 animals for each group. All data of IHC in mice were analyzed with Prism 7 (GraphPad Software). Data represent mean ± SEM. p values were generated by unpaired t test: **p < 0.01, ***p < 0.001, ****p < 0.0001; MPS-IIIA *IDSspSGSH-*IRES*-SUMF1*-treated versus MPS-IIIA GFP-treated; MPS-IIIA IDSspSGSH-IRES-SUMF1-treated versus WT GFP-treated. (B) Semiquantitative analysis of lysosome vacuolization on ultra-thin parietal cortex section stained with toluidine blue. The analysis was performed by analyzing 100 cells for each experimental group of mice. n = 5–7 animals for group. Data represent mean ± SEM. p values were generated by unpaired t test: ****p < 0.0001; MPS-IIIA IDSspSGSH-IRES-SUMF1-treated versus MPS-IIIA GFP-treated; MPS-IIIA IDSspSGSH-IRES-SUMF1-treated versus WT GFP-treated. (C) Quantitative analysis of GAG content (μg GAG/μg DNA) in whole-brain samples collected at LTP in MPS-IIIA mice i.c.v. injected with AAV9 encoding *IDSspSGSH*-IRES-*SUMF1*. Age-matched WT and MPS-IIIA mice i.c.v. injected with AAV9 encoding GFP were used as controls. N = 4–6 animals per group. Data represent mean ± SEM. ****p < 0.0001, one-way ANOVA followed by Tukey’s post hoc test. (D) Neuroinflammation was evaluated at LTP in MPS-IIIA mice injected with AAV9 encoding *IDSspSGSH*-IRES-*SUMF1* by immunostaining with anti-GFAP (astrogliosis) in paraffin sections from frontal cortex, parietal cortex, and lateral septum. Age-matched WT and MPS-IIIA mice injected with AAV9 encoding GFP were used as controls. Scale bars: 100 μm. Plots represent the quantification of the signal in the indicated area for each treatment group. n = 4–5 animals per group. Data represent mean ± SEM. p values were generated by unpaired t test: *p < 0.05, **p < 0.01; MPS-IIIA IDSspSGSH-IRES-SUMF1-treated versus MPS-IIIA GFP-treated; MPS-IIIA IDSspSGSH-IRES-SUMF1-treated versus WT GFP-treated. (E) MPS-IIIA mice and relative controls (WT) were tested at 6 and 9 months of age in the fear contextual conditioning test. MPS-IIIA mice show an age-dependent impairment, as evidenced by reduced freezing time, at only 9 months of age. Treatment with IDSspSGSH-IRES-SUMF1 fully rescued the freezing response in 9-month-old MPS-IIIA mice [age × group (F2/16 = 4.05; p = 0.03); age (F1/16 = 25.34; p = 0.0001); test phase (F1/16 = 84.5; p < 0.0001); test phase × group (F2/16 = 3.85; p = 0.04); test phase × age (F1/16 = 4.3; p = 0.053); age × test phase × group (F2/16 = 3.35; p = 0.06)]. *p < 0.05, Duncan post hoc analysis.

Abnormal lysosomal storage (including glycosaminoglycans [GAGs]) is associated with lysosomal compartment enlargement and neuroinflammation, both hallmarks of MPS-IIIA neuropathology.^7^^,^^24^ Immunostaining for lysosomal-associated membrane protein 1 (LAMP1) on brain sections of MPS-IIIA mice injected with AAV9-*IDSspSGSH*-IRES-*SUMF1* showed a significant reduction in the lysosomal compartment enlargement compared with control MPS-IIIA mice (Figure 3A). Such improvement was associated to a significant reduction in the abnormal vacuolization as shown by toluidine blue staining analysis (Figure 3B). Consistently, GAGs levels were also significantly reduced following *IDSspSGSH*-IRES-*SUMF1* treatment (Figure 3C). Next, to evaluate the inflammation levels in treated MPS-IIIA mice, brain sections were stained for GFAP, a marker of astrocytes. As expected, excessive astrocyte levels were seen in MPS-IIIA control mice (Figure 3D). Remarkably, the brain of MPS-IIIA mice treated with *IDSspSGSH*-IRES-*SUMF1* showed a strong reduction of GFAP signal as compared with control affected mice, indicating a striking reduction of inflammatory processes (Figure 3D).

Along with the severe manifestations, MPS-IIIA also shows mild somatic pathology characterized by GAG storage in several somatic tissues, particularly in the liver, which leads to increased mass (hepatomegaly). Being that the CSF is continuously absorbed and returned to the bloodstream, AAV9 vectors may travel from the CSF to the bloodstream and eventually reach the somatic tissues. The presence of such leakage was supported by the fact that following i.c.v. AAV9-mediated delivery of *IDSspSGSH*-IRES-*SUMF1* gene transfer, the livers of treated mice were efficiently transduced as shown by significant increased levels of vector genomes (Figure S3A) and a subsequent significant increase in SGSH enzymatic activity (Figure S3B). This increase was associated with higher levels of SGSH protein as well (Figure S3C). Importantly, quantitative measurement of GAGs in the liver of *IDSspSGSH*-IRES-*SUMF1*-treated MPS-IIIA mice showed an almost complete normalization of GAG content (Figure S3D). Together these results demonstrated the effectiveness of AAV9-mediated delivery of the enhanced version of SGSH with improved secretion and activation in rescuing both CNS and somatic pathology in MPS-IIIA mice.

### Treatment with *IDSspSGSH*-IRES-*SUMF1* Prevents the Memory Deficits Occurring in the Late Stage of MPS-IIIA Pathology

We next validated the impact of the i.c.v. AAV9-mediated delivery of *IDSspSGSH-*IRES*-SUMF1* on behavioral deficits that manifest in 6- and 9-months-old MPS-IIIA mice. Based on previous studies from our group, we evaluated exploratory behavior in the open field. At 6 months, MPS-IIIA male mice show a mild difference in the distance traveled, immobile time, and line crossing in the very first minutes of open field testing, as compared with WT animals (Figure S4C). This tendency was not evident at 9 months, likely due to the test-retest effects observed also in control animals,^25^ which did not allow to properly evaluate the rescuing efficacy of *IDSspSGSH-*IRES*-SUMF1* (Figures S4A–4C). Therefore, we tested the animals in a memory task that is extensively used to test contextual memory depending on the functional integrity of the medial temporal lobe, namely, the fear contextual conditioning test.^26^ Using this task, we identified, for the first time in MPS-IIIA mice, an age-dependent long-term memory impairment: 6-months-old MPS-IIIA mice show normal freezing time when they are exposed to a mild foot shock during training, as compared with control animals; similarly, exposure to the context (24 h after training) paired with the mild foot shock is sufficient to elicit freezing as well as in control mice (Figure 3E). This suggests that at this stage MPS-IIIA mice can form emotional contextual memories as well as WT animals. However, when the same test is repeated 3 months later, although there are no differences in basal freezing during training, on the testing day MPS-IIIA mice have impaired freezing response (Figure 3E). Interestingly, *IDSspSGSH-*IRES*-SUMF1* fully rescues the memory deficit in 9-month-old MPS-IIIA mice (Figure 3E). These behavioral effects in MPS-IIIA mice (both treated and untreated) cannot be explained as due to reduced immobility time (a possible correlate of reduced freezing time), because in the open field test both MPS-IIIA groups show similar levels of immobility time at this testing age. Considering the specificity of this test for memory deficits occurring in animal models of neurodegenerative diseases, these results have a high relevance for the validation of the neuronal dysfunctions correction in MPS-IIIA mice treated with AAV9-*IDSspSGSH-*IRES*-SUMF1*.

## Discussion

Here, we developed and tested an intra-CSF AAV-mediated gene transfer approach for the MPS-IIIA based on intrathecal delivery of AAV9 bearing a modified SGSH expression cassette (*IDSspSGSH-*IRES*-SUMF1*). Such expression cassette contained an SGSH bearing the signal peptide of IDS, which confers to the engineered enzyme the capability to be secreted at higher efficiency compared with the WT enzyme. Such modification resulted in improved biodistribution of the enzyme in the CNS of both small-animal (MPS-IIIA mice) and large-animal (WT pigs) models. Very recently, Chen et al.^12^ reported that intra-CSF delivery of AAV4 encoding a highly secreted variant of SGSH mutated in the mannose-6-phosphate (M6P) binding site was therapeutically superior to the intra-CSF AAV4-mediated delivery system based on the use of the WT enzyme. Both our and Chen et al.’s^12^ studies support the concept that enhancing SGSH secretion improves the distribution of enzyme in the CNS because of its enhanced cross-correction capability. Of note, whereas in the approach reported by Chen et al.^12^ the mutation of the M6P binding site makes SGSH uptake M6P independent, in our approach the mannose-6-phosphate receptor (M6PR)-mediated system, which underlies the uptake in disease-relevant cellular targets such as neurons and astrocytes, is preserved.^8^ Moreover, an important additional feature of the SGSH expression cassette used in our strategy is the insertion of the cDNA codifying for SUMF1, the enzyme responsible for the post-translational activation of sulfatases. Such modification synergistically acts together with enhanced secretion to further improve enzyme CNS biodistribution upon intra-CSF AAV9-mediated gene delivery. The finding that the additive effect of SUMF1 is not evident in the brain of pigs treated with WT enzyme (Figure 1A; Table S1) can be explained by the fact that the brain amount of SUMF1 becomes limiting for SGSH activation only in a context where SGSH has an enhanced cross-correction capability. Remarkably, SGSH enzyme biodistribution in the CNS was more efficient in the pig than in the MPS-IIIA mouse model, likely due to the disease state and different administration routes used.

Importantly, we demonstrated that the modified SGSH expression cassette we developed is therapeutically effective, being able to efficiently rescue CNS and somatic storage pathology, and improve memory impairment.

Despite that a direct demonstration of the therapeutic superiority of the modified SGSH construct containing both IDSsp and SUMF1 by a side-by side comparative analysis has not been provided, our data clearly show that using a modified SGSH expression cassette may have a potential therapeutic advantage. It is remarkable that the performance of MPS-IIIA mice treated with *IDSspSGSH-*IRES*-SUMF1* in the long-term memory task was undistinguishable from that of WT animals, thus suggesting that together with enhancing the biodistribution of the therapeutic enzyme, *IDSspSGSH-*IRES*-SUMF1* treatment also allows to obtain maximal improvement in functional tests.

Intra-CSF AAV9-mediated gene delivery using WT SGSH has previously been successfully tested in different preclinical animal models using vector doses similar to those used in our approach (i.e., ~4–5 × 10^12^ GCs/kg).^9^ In that study the authors also showed that reducing AAV9 vector dosage in MPS-IIIA mice below 4–5 × 10^12^ GCs/kg led to inefficient enzyme biodistribution, thus resulting in only mild neuropathology improvement. Here, we demonstrated that intra-CSF delivery of AAV9 bearing the modified *SGSH* expression cassette at doses of ~4–5 × 10^12^ GCs/kg led to a more efficient CNS enzyme biodistribution compared with WT SGSH both in small- and in large-animal models. Therefore, although we did not evaluate SGSH enzyme distribution at a dose below 4.5 × 10^12^ GCs/kg, our data suggested that by using our approach it might be possible to achieve and maintain therapeutic threshold levels of the enzyme throughout the CNS at reduced and, therefore, safer AAV9 vector dosages, thus making our approach potentially more attractive for clinical purposes.

Overall, the results presented here pave the way for developing gene delivery replacement protocols with enhanced therapeutic potential for the treatment of MPS-IIIA patients and also provide a proof of principle for potential use of this strategy for the treatment of other forms of LSDs caused by sulfatase deficiency.

## Materials and Methods

### Animals

#### Pigs

The animals enrolled were postnatal day (P) 30 castrated male WT large White × Duroc hybrids (*Sus scrofa*). These animals were transferred to our facility on the day of weaning (28th day after birth) and housed in multiple stalls with infrared heating lamps and monitored for general health. They were strictly monitored in order to rule out any pathology that may have affected the entire experiment. The study was conducted in accordance with the provisions of European Economic Community Council Directive 86/609 adopted by the Italian Government (DL 27/01/1992 No. 116) under the local approval of the Ethical Committee of the University of Bologna and under the approval of the Italian Ministry of Health.

#### Mice

P60 homozygous mutant [B6Cg-*Sgsh*^(mps3a/PstJ)^ mouse (*Sgsh*^−/−^) from Jackson Laboratory; phenotypically MPS-IIIA affected] and healthy (*Sgsh*^*+/+*^) C57BL/6 mice were used.^1^^,^^27^

Experiments were conducted in accordance with the guidelines of the Animal Care and Use Committee of Cardarelli Hospital in Naples and authorized by the Italian Ministry of Health.

### Recombinant AAV Vectors

Human SGSH (*SGSH*), *SGSH-*IRES*-SUMF1*, *IDSspSGSH*, *IDSspSGSH-*IRES*-SUMF1*, or *GFP* expression cassettes were cloned in single-stranded pAAV2.1-CMV-expression plasmids to generate the correspondent AAV serotype 9 viral vectors according to protocols established at AAV TIGEM Vector Core.

### AAV Administration

#### Pigs

All of the activities performed on the day of the injection have been thoroughly described by Sorrentino et al.^20^ For the intra cisterna magna (ICM) injection, the dorsal area of the neck was trimmed and surgically prepared, and the puncture of the cisterna magna was performed as previously described.^3^^,^^28^ In brief, animals received an intramuscular (IM) bolus of tiletamine-zolazepam (5 mg/kg) 10 min before induction; general anesthesia was achieved using sevoflurane with an induction mask. After orotracheal intubation and stabilization, venous access for fluid therapy was achieved from an auricular vein. Blood samples (6 mL) were collected through the femoral artery.

The dose of 4.5 × 10^12^ GCs/kg of viral vector in the volume range from 1.5 to 2.8 mL was injected slowly to avoid a sudden increase in intracranial pressure. Piglets were then placed in Trendelenburg position for 2 min in order to help the injected compound to spread toward the more rostral parts of the CNS. Animals were then monitored until complete recovery. During the following days, all animals were strictly monitored in order to rule out any possible side effect of the procedure and to evaluate any changes in behavior and consequentially in welfare.

#### Mice

Mice were anesthetized by intraperitoneal (IP) injection of ketamine (100 mg/kg) and xylazine (10 mg/kg), and placed on a stereotaxic instrument with a motorized stereotaxic injector. A midline incision was made to expose the bregma. A hole in the skull was made by a drill (anteroposterior +2.18 mm, mediolateral 0.6 mm, dorsoventral −1.7 mm). Recombinant AAV9 (rAAV9) vectors, 5.4 × 10^12^ GCs/kg, were injected in a volume of 10 μL into the lateral ventricles at a rate of 1 μL/min. After allowing the needle to remain in place for 5 min, the needle was slowly raised at a rate of 0.1 cm/min.

### Tissue Collection

#### Pigs

Animals were euthanized 1 month after injection with a single bolus (0.3 mL/kg) of Tanax, and total body perfusion with Dulbecco’s PBS was started. After median sternotomy, the right atrium was opened and the left ventricle was infused with 500 mL of warm Dulbecco’s PBS (+38°C) and 1,000 mL of cold Dulbecco’s PBS (+4°C); blood ejected from the right atrium was drained using a surgical aspirator. As far as CNS samples, collected tissues were the whole brain and cervical region of spinal cord. Dissection was performed using the technique previously described.^20^ Twelve coronal sections (0.5 cm) were cut covering the main regions of the brain (right and left hemispheres) and the cervical region of the spinal cord. Sections were then frozen in liquid nitrogen for biochemical analysis or fixed in 4% (w/v) paraformaldehyde in PBS for OCT embedding.

#### Mice

Mice were euthanized at 1, 6, and 7 months by injection with ketamine and xylazine before blood and CSF collection. CSF was collected by glass capillary inserted into the cisterna magna. For tissue collection, mice were intracardially perfused with PBS (pH 7.4), and brains were removed, divided into halves, and fixed for further analysis. The right half was sliced in five slices (A–E) and frozen in liquid nitrogen; the left half was sliced in two coronally anterior and posterior parts fixed in 4% (w/v) paraformaldehyde in PBS and embedded in paraffin. For toluidine blue staining analysis, a coronal slice derived from the left half of the brain was fixed in 4% paraformaldehyde, 25% glutaraldehyde in phosphate buffer for plastic embedding. Additionally, the left lateral lobe of the liver from the mice was fixed in 4% (w/v) paraformaldehyde in PBS and embedded in paraffin, and the right medial lobe of the liver of the mice was frozen in liquid nitrogen.

### SGSH Activity

Twenty main regions covering the entire CNS of injected pigs were dissected and homogenized with Tissue Lyser using 10 vol of H2O Milli-Q (700 μL). In mice, five different slices covering the entire brain and liver samples were homogenized separately with Tissue Lyser using 8 vol of H2O mQ. The SGSH activity was assayed by a 4-methylumbelliferone-derived fluorogenic substrate (4-MU; Moscerdam Substrates), following established protocols.^4^^,^^8^

### Immunoassay of SGSH

Immunoquantification of SGSH was performed in brain and liver samples of mice by using an ELISA technology. A total of 100 μL of anti-SGSH antibody R3074 from Shire (1 μg/mL) diluted in 0.05 M carbonate-bicarbonate buffer (pH 9.6; Sigma-Aldrich Japan, Tokyo, Japan) was used to coat multi-well plates at 37°C for 1 h. Afterward, samples or standards were incubated at 37°C for 1 h and then with horseradish peroxidase (HRP)-conjugated rabbit anti-HNS antibody R1315 (1:4,000) at 37°C for 1 h. The plate was then incubated with the HRP substrate, 3,3’,5,5’-tetramethylbenzidine (TMB) peroxidase substrate (1721066; Bio-Rad, Hercules, CA, USA), for 30 min at 37°C. This enzyme-substrate reaction was stopped using a stop buffer (2N of H_2_SO_4_), and the absorbance of each well was measured at the absorbance wavelength of 450 nm (Lm1) with a reference wavelength of 655 nm (Lm2) using a microplate reader (GloMax-Multi+ Detection System; Promega, Madison, WI, USA). The concentrations of HNS in samples were calculated using the HNS calibration curve in the same plate.

### Evaluation of AAV Vector Genome Copy Number in the CNS

Genomic DNA was extracted from mouse brain samples using a DNeasy Blood and Tissue Extraction kit (QIAGEN, Valencia, CA, USA). DNA concentration was determined by using a NanoDrop. Real-time PCR was performed on 100 ng of genomic DNA using a LightCycler SYBR green I system (Roche, Almere, the Netherlands). Amplification was run on a LightCycler 96 device (Roche) with standard cycles. The primers forward SGSH (5′-CATCCACTTTGCCTTTCTCTCCA-3′) and reverse SGSH (5′-TCAAAGCCTCCGTCATCCGC-3′) were used. A standard curve was generated, using the corresponding AAV vector plasmid pAAV2.1CMV-IDSspSGSH-IRES-SUMF1.

### GAG Quantification

Brain samples and liver samples were lysed in water by Tissue Lyser equipment. The lysates were then digested with Proteinase K, and extracts were clarified by centrifugation and filtration. GAG levels in brain extracts were determined using the Blyscan sulfated glycosaminoglycan kit (Biocolor, Carrickfergus, UK) with chondroitin 4-sulfate as the standard.

### Immunohistochemistry

IHC experiments were performed in 5-μm paraffin sections with BondRX Stainer following the standard protocol. The primary antibodies used were mouse anti-Human SGSH (Shire 2C7) 1:25 for immunofluorescence and 1:500 for IHC, rabbit anti-LAMP1 (ab24170; Abcam), rabbit anti-GFAP (ab7260; Abcam), and rabbit anti-NeuN (ab177487). Five-micrometer paraffin-embedded tissue sections were incubated overnight at 4°C with rabbit anti-GFAP (Z0334; Dako Cytomation). The detection system including secondary antibodies used for IHC was the Bond Polymer refine kit (DS9800; Leica) for the visualization of SGSH and LAMP1 signals. The secondary antibody used for the detection of GFAP signal was biotinylated universal antibody of Vectastain ABC kit (Vector Laboratories, Burlingame, CA, USA).

Bright-field sections were stained with 3,3-diaminobenzidine (Sigma-Aldrich) and counterstained with hematoxylin. The SGSH- and LAMP1-stained slides were scanned with Aperio ScanScope AT2 scanner. The whole digital slides were viewed and analyzed by ImageScope. The positive pixel count algorithm was selected and adjusted to cover each individual positive staining of LAMP1 for analysis. The data were presented as positivity, which was obtained from the following formula: Positivity (%) = positive area (pixels)/total analyzed area (pixels) × 100%.

The GFAP-stained slides were scanned with Hamamatsu Nanozoomer 2.0-RS scanner and viewed with NDP.view2. The total number of GFAP-positive signals was counted using the cell-counter program (ImageJ software) with a fixed threshold.

For immunofluorescence, secondary antibodies Donkey anti-mouse immunoglobulin G (IgG)-Alexa Fluor 488 (R37114; Thermo Fisher) and Goat anti-rabbit IgG-Alexa Fluor 568 (A-11011; Thermo Fisher) were used for the visualization. Stained slides were read and representative photos were taken with Nikon fluorescent microscope.

### Toluidine Blue Staining

Fixed samples of specific brain regions were post-fixed in 1% osmium tetroxide, dehydrated, and embedded in resin. One-micrometer sections were stained with 1% toluidine blue and examined by light microscopy. Ultra-thin sections from the selected region of cortex were cut and stained with 0.5% uranyl acetate.

### Behavioral Tests

Behavioral tests were carried out in a behavioral testing room maintained under constant light, temperature, and humidity. The mice were tested during daylight hours (between 9 a.m. and 6 p.m.). Before testing, animals were habituated to the testing room for at least 30 min. The same groups of animals were tested at 6 and 9 months of age. We performed the open field task, which in previous studies we have found to be impaired in adult MPS-IIIA mice. Additionally, we tested them in the contextual fear conditioning task, which allows to evaluate animal ability to learn and remember a Pavlovian association between a mild food shock and a specific context.

#### Open Field Task

The open field task was performed as previously described.^4^^,^^8^ Mice were placed in the middle of a Plexiglas arena with a masonite base (43 × 32 × 40 cm). Animals were left free to explore the device for 10 min. The distance traveled (m), the immobility time (s), and line crossing were recorded using a video camera (WV-BP330; Panasonic) hanging over the arena that was connected to a video-tracking system (ANY-maze; Stoelting, Chicago, IL, USA).

#### Contextual Fear Conditioning

Each mouse was trained in a conditioning chamber (30 cm × 24 cm × 21 cm; Ugo Basile) that had a removable grid floor and waste pan. The grid floor contained 36 stainless steel rods (3-mm diameter) spaced 8 mm center to center. When placed in the chamber, the grid floor contacted a circuit board through which scrambled shock was delivered. The shock intensity was 0.5 mA with a duration of 2 s, and it was presented three times and was associated to a context. After 24 h after training, mice were tested without foot shock but with the same context. Freezing behavior was defined as complete lack of movement, except for respiration, and scored with a video-tracking system (ANY-maze; Stoelting, USA).

### Statistical Analysis

Data of behavioral tests were expressed as mean ± SEM. Two-way ANOVA for repeated measures with the factor group as independent factor and trials or test phase as repeated-measures was performed to assess significance among multiple experimental groups and at different time points, followed by Duncan post hoc test when appropriate. A p value <0.05 was considered as statistically significant.

For the enzymatic activity, ELISA, vector copy number, and GAG analysis, the data were expressed as mean ± SEM. One-way comparisons were performed to calculate the significance among the experimental groups, followed by Tukey’s post hoc test. A p value <0.05 was considered as statistically significant.

Data of LAMP1, toluidine blue, and GFAP staining quantification were expressed as mean ± SEM. p values were generated by unpaired t test: *p < 0.05, **p < 0.01, ***p < 0.001.

## Author Contributions

N.C.S. co-supervised and performed research, analyzed data, and contributed to the writing of the entire manuscript. V.C., V.M., S.S., and N.T. performed research. E.N. maintained the mouse colony from TIGEM. N.R. and D.V. contributed with pig studies. Y.H. and N.L. conducted tissue sectioning, immunohistochemistry IHC, and data analysis from Shire. S.L.K. and V.W.C. supervised project direction, study designs, and data interpretation from Shire. M.D.R. performed and analyzed behavioral data. E.D.L. conceived the behavioral studies, contributed to the analysis of behavioral data, supervised behavioral studies, and contributed to the writing of the manuscript (behavioral results). A.F. designed and supervised research, and wrote the manuscript.

## Conflicts of Interest

Y.H., N.L., S.L.K., and V.W.C. are employees and stockholders of Shire. The other authors declare no competing interests.

## Supplemental Information

Document S1. Figures S1–S4 and Table S1

Document S2. Article plus Supplemental Information
