# Supplementary material for: Enhancing the Therapeutic Potential of Sulfamidase for the Treatment of Mucopolysaccharidosis IIIA
Source: Mol Ther Methods Clin Dev. 2019 Oct 28;15:333–42. doi: 10.1016/j.omtm.2019.10.009 (PMC6881609; doi:10.1016/j.omtm.2019.10.009)
Supplement: Document S1. Figures S1–S4 and Table S1 [file mmc1.pdf]

OMTM, Volume *15*

Supplemental Information

Enhancing the Therapeutic Potential of Sulfamidase for the Treatment of Mucopolysaccharidosis IIIA

Nicolina Cristina Sorrentino, Vincenzo Cacace, Maria De Risi, Veronica Maffia, Sandra Strollo, Novella Tedesco, Edoardo Nusco, Noemi Romagnoli, Domenico Ventrella, Yan Huang, Nan Liu, Susan L. Kalled, Vivian W. Choi, Elvira De Leonibus, and Alessandro Fraldi

Supplementary Figure 1

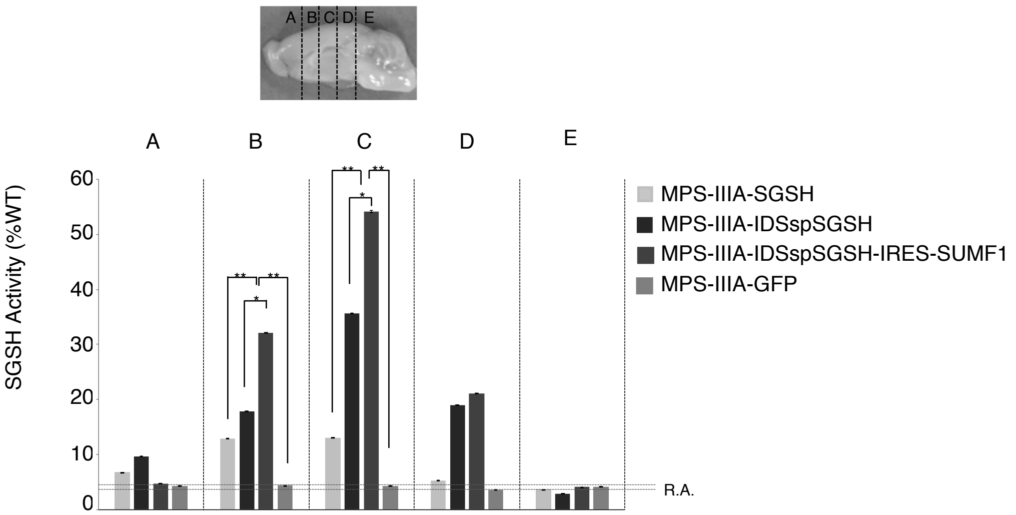

Supplementary Figure 1. Sulfamidase activity in the brain of MPS-IIIA mice injected with AAV9 vectors carrying different SGSH expression cassettes

P60 MPS-IIIA mice were intra-CSF injected (via lateral ventricle administration: ICV) with 5.4×10^{12} GC/Kg of AAV9 encoding under the CMV promoter the following expression cassettes: *GFP*, *SGSH* WT, *IDSspSGSH*, or *IDSspSGSH*-IRES-*SUMF1*. The brain and the first region of the spinal cord of treated mice was divided in five slices (A-E) covering the main representative area of the CNS (A: olfactory bulb and prefrontal cortex, B: frontal cortex, lateral septum and basal ganglia regions, C: parietal cortex, hippocampus, striatum, thalamus, D: occipital cortex, pons, hippocampus; E: cerebellum, medulla oblongata, cervical region of spinal cord). One month after injection sulfamidase activity was measured in these areas and expressed as the percentage of the activity found in control GFP-treated WT mice. N = 3-4 animals per group. Data represent mean ± SEM. *$P<0.05$, **$P<0.01$ MPS-IIIA-IDSspSGSH-IRES-SUMF1VS MPS-IIIA-GFP, MPS-IIIA-IDSspSGSH-IRES-SUMF1 VS MPS-IIIA-IDSspSGSH, MPS-IIIA-IDSspSGSH-IRES-SUMF1 VS MPS-IIIA-SGSH. One-way ANOVA followed by Tukey's post hoc test.

Supplementary Figure 2

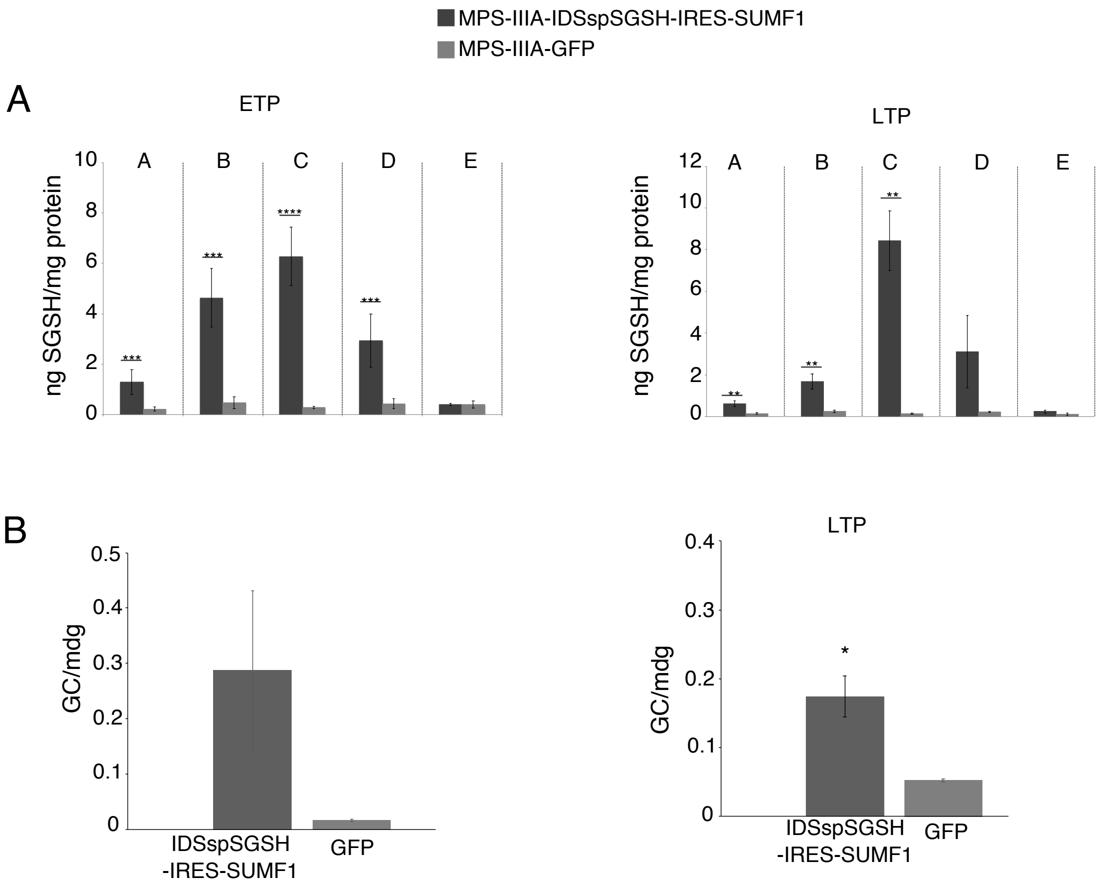

Supplementary Figure 2. Sulfamidase protein and vector copy numbers quantitation in the brain of MPS-IIIA mice injected with AAV9 bearing the IDSspSGSH-IRES-SUMF1 transgene

(A) Sulfamidase protein was immuno-quantified by ELISA and expressed as ng of SGSH/mg protein in the five CNS slices (A-E; as described in the supplementary figure 1) of the indicated experimental groups of mice at ETP and LTP. Age-matched WT and MPS-IIIA mice ICV injected with AAV9 encoding for GFP were used as control. Data represent mean ± SEM. N= 5-7 animals for each group. **P<0.01, ***P<0.001, ****P<0.0001 VS MPS-IIIA-GFP. One-way ANOVA followed by Tukey's post hoc test. (B) Vector genome copy number (expressed as GC/mouse diploid genome; mdg) were measured in the whole brain samples from MPS-IIIA mice ICV injected with AAV9 encoding IDSsp*SGSH*-IRES-*SUMF1* and age-matched MPS-IIIA mice ICV injected with AAV9 encoding GFP at ETP and LTP. Data represent mean ± SEM. N= 5-7 animals for each group. *P<0.05, Student T-test.

Supplementary Figure 3

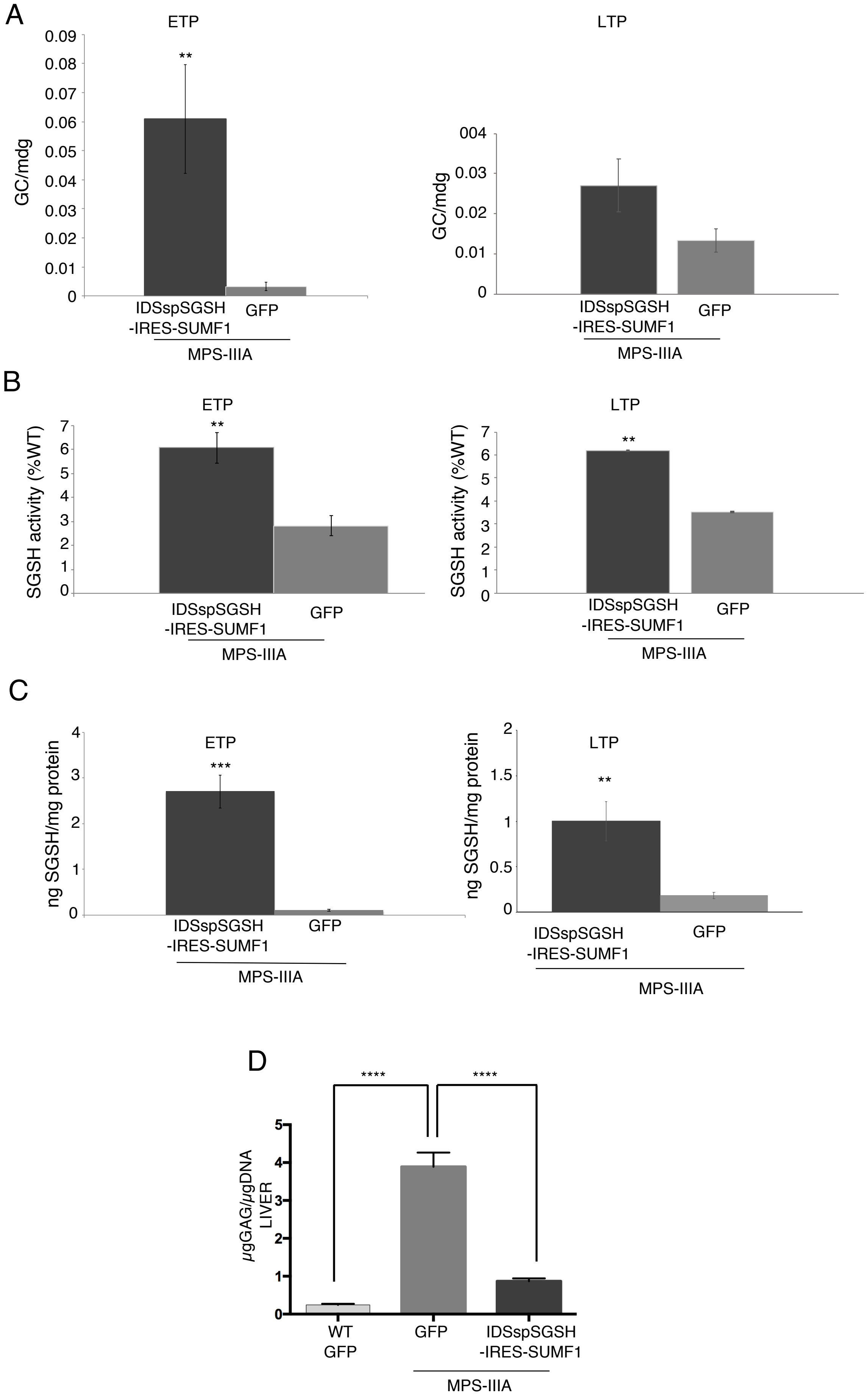

Supplementary Figure 3. Liver transduction in MPS-IIIA mice injected with AAV9 bearing IDSspSGSH-IRES-SUMF1 transgene

(A-C) Vector genome copy number (expressed as GC/mouse diploid genome; mdg) (A), sulfamidase activity (expressed as the percentage of WT sulfamidase activity) (B) and ELISA immunoquantification of the sulfamidase protein (expressed as ng of SGSH/mg protein) (C) were measured in liver samples from MPS-IIIA mice ICV injected with AAV9 encoding IDSsp*SGSH*-IRES-*SUMF1* and age-matched WT and MPS-IIIA mice ICV injected with AAV9 encoding GFP at ETP and LTP. Data represent mean ± SEM. N= 6-8 animals for each group. **P<0.01, ***P<0.001. *vs* MPS-IIIA GFP-treated. Student T-test. (D) Quantitative analysis of GAG content (µgGAG/µgDNA) in liver samples collected at LTP in MPS-IIIA mice ICV injected with AAV9 encoding *IDSspSGSH*-IRES-*SUMF1*. Age-matched WT and MPS-IIIA mice ICV injected with AAV9 encoding GFP were used as controls. N = 6 animals per group. Data represent mean ± SEM; ****P<0.0001. One-way ANOVA followed by Tukey's post hoc test.

Supplementary Figure 4

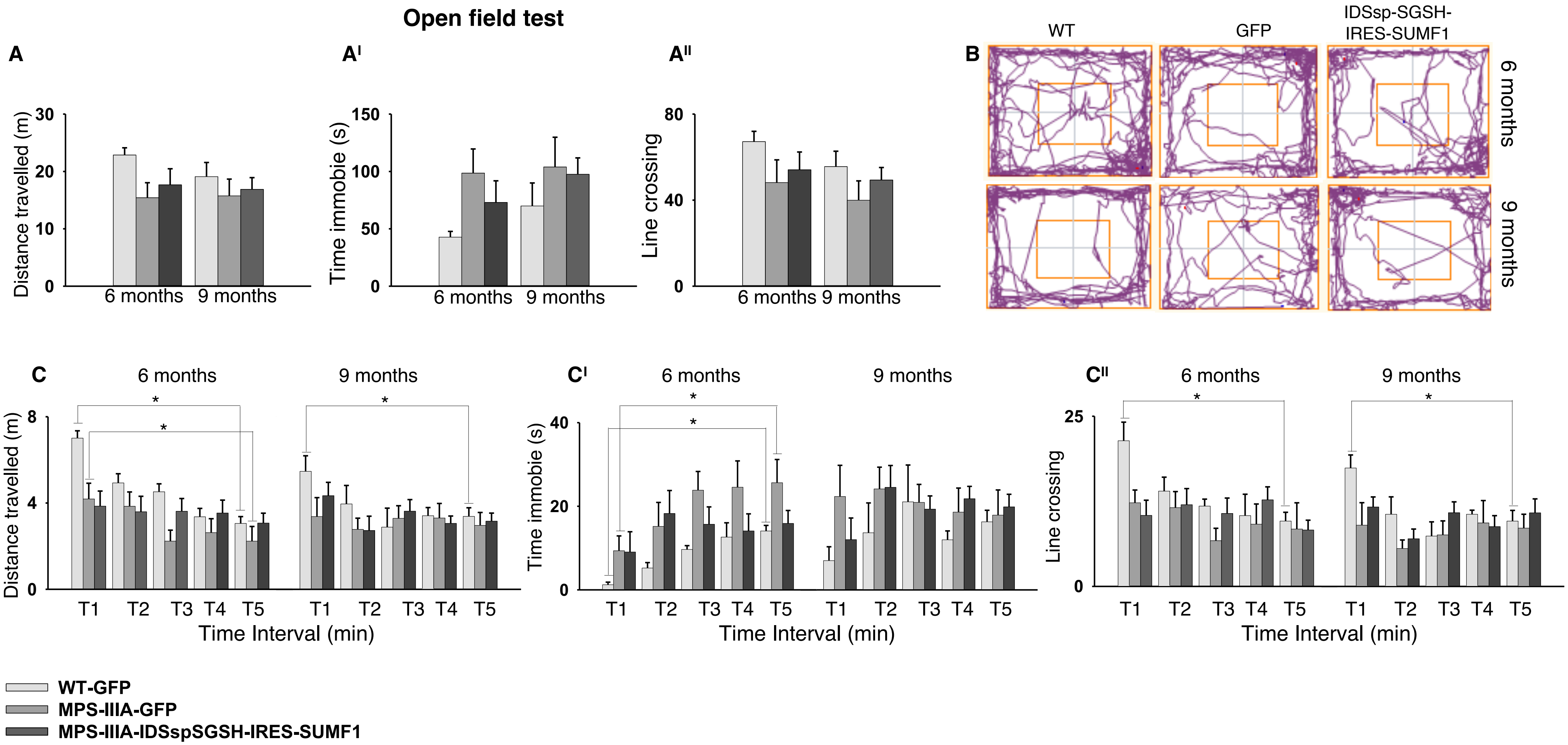

Supplementary Figure 4. Assessment of exploratory activity in MPS-IIIA mice injected with AAV9 bearing IDSspSGSH-IRES-SUMF1 transgene.

MPS-IIIA mice and relative controls (WT) were tested at 6 and 9 months of age in the open field test. (A- AII) There were no significant differences between groups at any of the testing age in the total distance travelled (m) [Group (F2/16=1.16; p=0.22); Distance (F1/16=0.65; p=0.43); Group x Distance x Age (F2/16=0.45; p=0.64)] (A), total number of line crossings [Group (F2/16=1.94; p=0.17); Line crossing (F1/16=1,77; p=0.20); Group x Line crossing x Age (F2/16=0.24; p=0.78)] (AI) and total immobility time (sec) [Group (F2/16=1.46; p=0.25); Immobility time (F1/16=2.31; p=0.14); Group x Immobility time x Age (F2/16=0.12; p=0.88)] (AII). (C-CII) A deeper analysis of the results considering 1 min time intervals (T1-T5) evidenced that at 6 months of age MPS-IIIA mice, as compared to WT littermates, showed reduced distance travelled [Time interval (F4/64=17.9; $p<0.0001$); Time intervals x Group (F4/64=3.16; p=0.004), Age x Time intervals x Group (F4/64=2.6; p=0.01)] (C), increased immobility time [Time intervals (F4/64=2.4; p=0.05); Group x Age x Time interval (F8/64=2.04; p=0.05)] (CI) and reduced line crossing frequency [Time interval (F4/64=6.8; p=0.0001); Group x Time intervals (F4/64=2.66; p=0.01)] (CII) mainly present in the very first minute of the task; these behavioral defects, however, were not anymore detectable at 9 months of ageing probably due to a test-retest habituation effect observed in WT animals [distance, age x time interval (F8/64=3.73; p=0.0085); time immobile, age x time intervals (F4/64=1.61; p=0.05), line crossing, age x time intervals (F4/64=2.4; p=0.05)]. Representative track-plots of the trajectory in the open field (B). * $p<0.05$, Duncan post hoc analysis.

Supplementary Table 1

Pvalue of SGSH activity in CNS areas of wild type pigs				
CNS regions	IDSsp-SGSH-IRES-SUMF1 vs IDSspSGSH	IDSsp-SGSH-IRES-SUMF1 vs SGSH-IRES-SUMF1	IDSsp-SGSH-IRES-SUMF1 vs SGSH	IDSsp-SGSH-IRES-SUMF1 vs PBS
Frontal cortex	ns	ns	*	**
Accumbens	**	**	****	****
Parietal ccortex	ns	**	***	****
Amigdala	ns	*	**	***
Hypotalamus	ns	**	*	**
N.caudatus	ns	*	*	***
Putamen	ns	**	*	***
Sub.Nigra	ns	*	*	**
Stria	ns	*	*	***
Hippocmpus	ns	**	**	***
Thalamus	*	***	**	****
C.callosum	*	****	****	****
Subcallosus	*	**	***	****
N.Pontis	ns	ns	ns	**
Culliculli	*	**	*	***
Occipital cortex	ns	**	**	***
Cerebellum	ns	*	*	***
Pons	**	*	**	****
Med. Ablong.	ns	*	ns	***
Spinal cord	ns	ns	*	***

P ≤0,05; ** P ≤0,01; *** P ≤0,001; **** P ≤0,001; ns: not significant.
No significant differences of SGSH activity in CNS regions among SGSH-IRES-SUMF1 VS SGSH experimental groups.
